# Distinct trajectories of multimorbidity in primary care were identified using latent class growth analysis^☆^

**DOI:** 10.1016/j.jclinepi.2014.06.003

**Published:** 2014-10

**Authors:** Vicky Y. Strauss, Peter W. Jones, Umesh T. Kadam, Kelvin P. Jordan

**Affiliations:** a Arthritis Research UK Primary Care Centre, Keele University, the Institute of Primary Care and Health Sciences, Keele, Staffordshire ST5 5BG, United Kingdom; b Centre for Statistics in Medicine, Nuffield Department of Orthopaedics, Rheumatology and Musculoskeletal Sciences, Botnar Research Centre, University of Oxford, Windmill Road, Oxford OX3 7LD, United Kingdom; c The Health Service Research Unit, the Institute for Science & Technology in Medicine, Innovation Centre 1, Keele University, Staffordshire ST5 5BG, United Kingdom

**Keywords:** Latent class growth analysis, Medical records, Primary health care, Multimorbidity, Comorbidity, Longitudinal studies

## Abstract

**Objectives:**

To investigate the use of latent class growth analysis (LCGA) in understanding onset and changes in multimorbidity over time in older adults.

**Study Design and Setting:**

This study used primary care consultations for 42 consensus-defined chronic morbidities over 3 years (2003–2005) by 24,615 people aged >50 years at 10 UK general practices, which contribute to the Consultations in Primary Care Archive database. Distinct groups of people who had similar progression of multimorbidity over time were identified using LCGA. These derived trajectories were tested in another primary care consultation data set with linked self-reported health status.

**Results:**

Five clusters of people representing different trajectories were identified: those who had no recorded chronic problems (40%), those who developed a first chronic morbidity over 3 years (10%), a developing multimorbidity group (37%), a group with increasing number of chronic morbidities (12%), and a multi-chronic group with many chronic morbidities (1%). These trajectories were also identified using another consultation database and associated with self-reported physical and mental health.

**Conclusion:**

There are distinct trajectories in the development of multimorbidity in primary care populations, which are associated with poor health. Future research needs to incorporate such trajectories when assessing progression of disease and deterioration of health.

## 1 Introduction

**What is new?**

**Key findings**
• Five distinct trajectories of multimorbidity for chronic diseases over time in older adults were identified and validated: those who had no recorded chronic problems (40%), those who developed a first chronic morbidity over 3 years (10%), a developing multimorbidity group (37%), a group with increasing number of chronic morbidities (12%), and a multi-chronic group with many chronic morbidities (1%). These trajectories have different self-reported physical and mental health profiles.

**What this adds to what was known?**
• Heterogeneity in the accumulation of multimorbidity over time in primary care in older adults can be summarized via latent class growth analysis into five distinct longitudinal trajectories of chronic multimorbidity. These patterns of multimorbidity are associated with physical and mental health.

**What is the implication and what should change now?**
• Future research in multimorbidity needs to recognize that older adults develop chronic multimorbidity at different rates over time. The importance of differing multimorbidity trajectories also needs to be recognized when studying unnoticed multimorbidity patterns, progression of disease, and deterioration of health.
• Recognition of multimorbidity pathways may aid clinicians move away from a single disease management approach in older adults.

It is estimated that over half of people aged ≥65 years have multiple chronic morbidities [1], [2], [3], [4], [5]. Multimorbidity in older adults is linked with worse health outcomes including mortality, worse physical function status, and increased health care usage [6], [7], [8], [9]. Care packages recommended by disease-specific guidelines may be difficult to operate in people with multimorbidity and may even be potentially harmful [9], [10].

Three recent systematic reviews on multimorbidity have highlighted a need to better understand the development of multimorbidity [9], [11], [12]. This information may help clinicians understand reasons for poorer health in certain people and aid their management. It may also aid researchers to assess the effect of multimorbidity on long-term outcome, identify prognostic factors, and develop interventions. Previous studies of multimorbidity have often been based on self-reported, which may be less accurate than primary care records [13], [14]. In the United Kingdom, primary care is commonly the point of entry into the health care system for people with a new symptom or illness and the vast majority of the population is registered with a general practitioner (GP). Primary care records should, therefore, be a comprehensive indicator of morbidities for which health care is sought [15], [16].

Approaches to measuring multimorbidity have included counting cooccurring morbidities or prescriptions [4], [5], [8], [12], [17], [18], [19], [20], [21], [22], [23], [24], [25], grouping morbidities using statistical methods such as cluster analysis and latent class analysis [26], [27], [28], and multimorbidity indices that are an aggregated score based on weighting-specified morbidities in terms of outcomes such as risk of mortality or disability [12], [29], [30], [31], [32]. These approaches have focused on a list of arbitrarily chosen morbidities (typically 6–25 [33]) and, therefore, do not cover the wider picture of multimorbidity in the older population. Existing primary care multimorbidity indices require clinical assessment of the patient [29], [34] or have limitations in comparing health impact of multimorbidity between people [29], [35], [36], [37].

Studies have generally measured multimorbidity at a single point or period in time, and thus ignored changes in multimorbidity over time. Two prospective studies found that changes in comorbidity significantly increased long-term mortality risks in people with breast cancer [38] and heart attack [39]. A Dutch study found that developing multimorbidity over time was associated with poorer physical functioning, but there was a little association with psychological health status [40]. An American study based on seven self-reported chronic morbidities identified wide variation in the rate of increasing multimorbidity over 5–6 years [41]. Recently, a general practice database study identified the accumulation of nine chronic cardiovascular morbidities over time [15]. However, none of these studies has attempted to describe the accumulation of morbidities based on a large number of diverse conditions and to identify whether there are distinct trajectories of multimorbidity over time using primary care consultations. Identifying different multimorbidity trajectories will help understanding the development of multimorbidity and aid prognostic studies identifying people at risk of more severe multimorbidity trajectories and associated adverse outcomes such as poor quality of life.

Latent class growth analysis (LCGA) has previously been used, for example, to group (cluster) people into distinct pathways of self-reported pain in adolescents [42] and childhood physical aggression [43]. However, it has not previously been applied to measure multimorbidity trajectories in routinely recorded health care data. The objectives of this study were (1) to use LCGA to identify distinct multimorbidity trajectories based on primary care consultations for chronic problems over time by older adults and (2) to test the existence of these trajectories in a second consultation database and their association with self-reported health status.

## 2 Methods

### 2.1 Phase I: developing trajectories of multimorbidity

The data set used in phase I contained anonymized primary care medical records for the 3-year period (2003–2005) from 10 general practices in North Staffordshire, UK, which contribute to the Consultations in Primary Care Archive (CiPCA). Ethical approval for CiPCA was obtained from the North Staffordshire Local Research Ethics Committee. The practices involved undergo an annual cycle of assessment, feedback, and training in quality of morbidity recording [44]. CiPCA has been shown to give comparable prevalence figures to other UK general practice consultation databases [45]. People, who were aged ≥50 years, were included and permanently registered at the practices during the 3-year period.

One hundred eighty-eight morbidities have previously been classified based on each of four criteria: chronicity, time course (one-off, recurrent, progressive, or permanent), extent of health care use, and patient impact using a consensus exercise involving 44 GPs [46]. These 188 included the 56 most commonly recorded morbidities in UK primary care, 18 selected on the basis of a previous study in the elderly, and 114 randomly selected morbidities. Previous studies have shown that over 80% of older English and Dutch general practice consulters had consulted for at least one of these morbidities during the course of a year and they had worse self-reported physical health than those who consulted for other morbidities. Furthermore, those who consulted for morbidities classified into more severe categories of the four criteria reported the worst health [47], [48]. In the present study, an initial latent class analysis using these criteria, validated by a clinical consensus exercise with eight GPs, determined that 42 of these morbidities (listed in Appendix at www.jclinepi.com) could be classified as being chronic in nature, generally progressive, and have lasting impact on people and health care use [49]. These 42 morbidities were identified in the primary care records of the included people using Read Codes, a common hierarchical method of recording morbidity in UK primary care. The morbidities were collated at the third level of the Read Code hierarchy and so included all lower level codes representing more detailed description of the morbidities. For example, diabetes mellitus (C10) includes C10E “Type 1 Diabetes Mellitus” and C10F “Type 2 Diabetes Mellitus.” In addition, we reviewed consultations recorded only at the higher second level as to whether they could also refer to any of the included morbidities. For example, G2, G3, and H3 were also used for high blood pressure, ischemic heart disease, and chronic bronchitis, respectively. The 3-year period was split into 6-month periods. For the first period, the number of the 42 chronic morbidities recorded in a patient's primary care record was determined. For each successive period, the cumulative number of the 42 morbidities consulted for in all the previous periods and new morbidities was determined. A person's full multimorbidity trajectory hence comprised the count of recorded chronic morbidities for six periods.

LCGA models were fitted starting with a one-cluster model, assuming that all subjects have the same trajectory, and then successively increasing the number of clusters until most of the heterogeneity in the data was explained [50], [51]. Counts in each period were assumed to be Poisson distributed. Quadratic growth curves were applied for all clusters identified within the LCGA models. For each model, people were assigned to the cluster where their posterior probability of membership was highest (the maximum probability assignment rule). Hence, people could only belong to one cluster. Both the Bayesian Information Criterion (BIC) and the adjusted likelihood ratio test proposed by Lo, Mendell, and Rubin (LMR) were used to determine optimal number of clusters [52]. The optimal model is that which has the lowest BIC value while the LMR test assesses whether adding one further cluster significantly improves the model fit.

The following criteria were also assessed: (1) presence of distinct cluster-specific trajectories, (2) inclusion of at least 1% of the people in the data set in the smallest cluster, and (3) allocation of people to their cluster with a high likelihood of being in that cluster based on the cluster-specific average posterior probabilities (AvePPs). Cluster-specific AvePPs are based only on the people assigned to that cluster and are calculated as the mean of their posterior probabilities. An AvePP greater than 0.7 was taken to suggest clear classification of people into clusters [53]. All LCGA models were estimated using Mplus V6.1 (Muthén & Muthén, Los Angeles, CA) with 1,000 randomly generated starting values [54].

Demographic characteristics including age group (50–64, 65–74, and >75 years), gender, and deprivation status, which have been shown to be associated with increasing morbidity [2], [5], [8], [11], [12], were compared between clusters using chi-square tests via SPSS V22. People were allocated a neighborhood deprivation score based on the England Index of Multiple Deprivation 2004 [55], with those scoring below the lowest quintile defined as the most deprived group and those scoring above the upper quintile as the least deprived. Deprivation score relates to the neighborhood in which a person lives and will vary between patients registered with the same practice.

### 2.2 Phase II: testing the trajectories

We used two cohorts of the North Staffordshire Osteoarthritis Project (NorStOP), a previously conducted general investigation of the health of older people, to test the clusters of multimorbidity trajectories [56]. This contained two parts: (1) external validation that assessed whether NorStOP participants showed a good fit to the multimorbidity trajectories identified in CiPCA and (2) comparison of the multimorbidity trajectories with self-reported health status.

NorStOP was approved by the North Staffordshire Local Research Ethics Committee. All people aged ≥50 years registered with six general practices were sent a postal questionnaire containing general health, sociodemographic, and pain-related questions, and consent was requested to link survey data to medical records [56]. Three of the six practices also contributed to the CiPCA analysis; therefore, only respondents from the other three practices, who consented to record review, were included in the validation phase. Consultation data were available for the period 2 years before the survey to 1 year afterward (January 2000–March 2002). The questionnaire included the Short Form-12. From this, a Physical Component Summary (PCS) and a Mental Component Summary (MCS) score were derived. These are weighted summary scores with a general population mean of 50 (standard derivation [SD]: 10), with lower scores indicating worse health [57].

The posterior probabilities of membership of each cluster identified in phase I of the study were estimated in Mplus [54], and participants were allocated to the cluster for which they had the highest posterior probability. The AvePPs were then used to test whether NorStOP respondents had a good fit to the identified trajectories of multimorbidity, again using values >0.7 to indicate clear allocation.

Differences in mean PCS and MCS scores among the clusters were tested using analysis of variance with a predetermined linear contrast via SPSS V22 which assumed that the cluster without a chronic morbidity consultation during the 3 years had the highest (best) mean PCS and MCS scores, and the means then decreased with increasing severity of multimorbidity trajectories. Finally, this trend was further assessed by adjusting for age, gender, and deprivation using multiple linear regression to ensure that it was not due to differences on these characteristics.

## 3 Results

### 3.1 Phase I

Of the 27,410 people aged ≥50 years who were registered at CiPCA practices at the end of 2005, 24,615 (90%) were fully registered during 2003–2005 and hence were included in the analysis. People included in the analysis were more likely to be older and female (*P* < 0.001).

The five-cluster model provided the best fit based on the smallest BIC value (Table 1), and the LMR test comparing the six- with the five-cluster model returned a nonsignificant result. People generally displayed high posterior probabilities of belonging to their assigned clusters (AvePP ranging from 0.76 to 0.96 across the five clusters).

**Table 1:** Statistical assessment of the optimal number of clusters from latent class growth analysis models based on counts of chronic morbidities across the six periods

Number of clusters	Log likelihood	Number of parameters	BIC	LMR significance level
1	−179,683	3	359,396	—
2	−136,179	7	272,429	***
3	−130,670	11	261,451	***
4	−129,424	15	258,999	***
5	−129,273	19	258,739	***
6	−129,266	23	258,766	

*Abbreviations*: BIC, Bayesian Information Criterion; LMR, Lo, Mendell, and Rubin likelihood ratio test.

***P < 0.01.

**Fig. 1:**
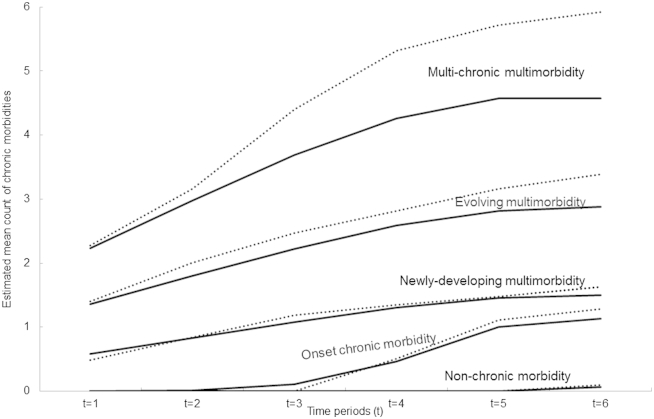
Clusters of chronic multimorbidity trajectories over time in Consultations in Primary Care Archive (CiPCA; solid lines) and North Staffordshire Osteoarthritis Project (NorStOP; dotted lines). The solid lines represent the estimated mean curves of multimorbidity profiles for the five clusters based on CiPCA. The dotted lines represent the mean of observed morbidity counts at each time point of NorStOP participants assigned to each of the clusters. “Non-chronic morbidity” cluster included people who did not have any chronic morbidity; “Onset chronic morbidity” cluster included those who developed a first chronic morbidity; “Newly-developing multimorbidity” cluster included those who developed multimorbidity lately; “Evolving multimorbidity” cluster included those who progressed from one chronic morbidity to multimorbidity; “Multi-chronic multimorbidity” cluster included those who started with multimorbidity and developed further morbidities.

Assessment of the cluster-specific trajectories suggested these could be described as non-chronic morbidity (40%), onset of chronic morbidity (10%), newly-developing multimorbidity (37%), evolving multimorbidity (12%), and multi-chronic multimorbidity (1%; Table 2, Fig. 1). The non-chronic cluster was dominated by people (93%) without any recorded chronic morbidity over the 3 years. People in the onset of chronic morbidity cluster (10%) did not have any recorded chronic morbidity in the first two periods and had a first record of a chronic disease (eg, high blood pressure, osteoarthritis, deafness, or chronic bronchitis) from the third period. The newly-developing multimorbidity cluster (37%) was characterized by people who progressed from zero or one chronic morbidity at the first period to two or three by the sixth period. The most common trajectories included people who started with hypertension in the first period and developed osteoarthritis, diabetes mellitus, and/or pure hypercholesterolemia over the six periods.

**Table 2:** Characteristics of the five clusters of multimorbidity trajectories

Characteristic	Non-chronic morbidity, *n* (%)	Onset of chronic morbidity, *n* (%)	Newly-developing multimorbidity, *n* (%)	Evolving multimorbidity, *n* (%)	Multi-chronic multimorbidity, *n* (%)	Significance level
	9,843 (40)	2,371 (10)	9,160 (37)	2,910 (12)	331 (1)	
Age (yr)						
50–64	7,270 (74)	1,327 (56)	4,287 (47)	930 (32)	78 (24)	***
65–74	1,677 (17)	593 (25)	2,815 (31)	1,114 (38)	134 (40)	
>75	896 (9)	451 (19)	2,058 (22)	866 (30)	119 (36)	
Gender						
Female	5,300 (54)	1,272 (54)	5,166 (56)	1,611 (55)	182 (55)	***
Male	4,543 (46)	1,099 (46)	3,994 (44)	1,299 (45)	149 (45)	
Deprivation						
Most deprived	1,966 (20)	464 (20)	1,835 (20)	659 (23)	69 (21)	*
Mid deprived	5,758 (59)	1,406 (59)	5,409 (59)	1,628 (54)	184 (56)	
Least deprived	2,116 (21)	501 (21)	1,913 (21)	623 (21)	78 (23)	

*P < 0.1; ***P < 0.01.

Two clusters had multimorbidity profiles which showed increasing numbers of chronic morbidities over the six periods (evolving multimorbidity and multi-chronic multimorbidity). By the sixth period, all the people in these clusters were multimorbid and tended to have at least two of the following conditions: hypothyroidism, diabetes mellitus, hypertension, angina, heart diseases, and osteoarthritis, plus other chronic diseases. In contrast to the evolving cluster, those in the multi-chronic cluster were most likely to start with multimorbidity (81% vs. 54%) and continued to have higher counts of morbidities in the following periods.

The younger age groups were more likely to be in the non-chronic cluster with a higher proportion of the older age groups in the evolving and multi-chronic clusters. Females were slightly more likely to develop or start with multimorbidity than males. The neighborhood deprivation status was borderline significantly associated with cluster membership (*P* = 0.05), with people in the most deprived areas more likely to be in the evolving multimorbidity cluster.

### 3.2 Phase II

A total of 8,904 people at the three practices had been mailed the baseline NorStOP questionnaire. Of those people, 6,347 (71%) responded of whom 4,532 (71%) consented to medical record review and were included in the data set for external validation. NorStOP respondents had high posterior probabilities of membership of assigned clusters (AvePP ranging from 0.75 to 0.97 across the five clusters) and low probabilities of belonging to the other clusters, indicating a good fit (Table 3). The five multimorbidity clusters (Fig. 1) revealed similar patterns of mean counts of chronic morbidities in each period in NorStOP responders to those in CiPCA.

**Table 3:** Average posterior probabilities by assigned cluster for the testing population

Assigned cluster	*N* (%)	Average posterior probabilities for each cluster (95% confidence interval)				
		Non-chronic morbidity	Onset chronic morbidity	Newly-developing multimorbidity	Evolving multimorbidity	Multi-chronic multimorbidity
Non-chronic morbidity	1,743 (34)	**0.97 (0.96, 0.97)**^a^	0.03 (0.03, 0.03)	0 (0, 0)	0 (0, 0)	0 (0, 0)
Onset of chronic morbidity	461 (11)	0.01 (0, 0.01)	**0.79 (0.78, 0.80)**^a^	0.20 (0.20, 0.21)	0 (0, 0)	0 (0, 0)
Newly-developing multimorbidity	1,759 (41)	0 (0, 0)	0.02 (0.01, 0.02)	**0.91 (0.91, 0.92)**^a^	0.07 (0.07, 0.08)	0 (0, 0)
Evolving multimorbidity	544 (13)	0 (0, 0)	0 (0, 0)	0.16 (0.15, 0.18)	**0.80 (0.79, 0.81)**^a^	0.04 (0.03, 0.04)
Multi-chronic multimorbidity	25 (1)	0 (0, 0)	0 (0, 0)	0 (0, 0)	0.25 (0.18, 0.33)	**0.75 (0.67, 0.82)**^a^

a The values in bold indicate the average posterior probabilities for each assigned cluster.

Significant differences between clusters were found for age and gender with similar patterns as found in CiPCA. People in the most deprived areas were more likely to be in the evolving and multi-chronic multimorbidity clusters. We assumed a priori that the mean PCS and MCS scores should decrease (worsen) across multimorbidity clusters in the following order: non-chronic morbidity, onset of chronic morbidity, newly-developing multimorbidity, evolving multimorbidity, and multi-chronic multimorbidity. There were significant decreasing trends for both mean PCS and MCS scores over the clusters supporting the assumed order (Table 4). NorStOP respondents who were allocated to the non-chronic cluster had the highest (best health) PCS and MCS scores. The mean baseline PCS score for this cluster (46.6, SD: 10.9) was almost twice that for the multi-chronic multimorbidity cluster (26.4, SD: 8.4). These differences remained after adjustment for age, gender, and deprivation.

**Table 4:** Self-reported health status and demographics for the North Staffordshire Osteoarthritis Project population, stratified by cluster

Characteristic	Non-chronic morbidity	Onset chronic morbidity	Newly-developing multimorbidity	Evolving multimorbidity	Multi-chronic multimorbidity	Significance level
*n*	1,743	461	1,759	544	25	
PCS mean (SD)	46.6 (10.9)	40.7 (11.7)	38.1 (12.0)	31.8 (10.6)	26.4 (8.4)	***
PCS mean difference (95% CI)^a^	0	−4.9 (−6.1, −3.7)	−7.1 (−7.9, −6.3)	−12.7 (−13.8, −11.5)	−18.0 (−22.6, −13.3)	
MCS mean (SD)	50.5 (10.5)	49.1 (11.2)	48.8 (11.4)	47.2 (12.3)	45.2 (11.6)	***
MCS mean Difference (95% CI)^a^	0	−1.6 (−2.8, −0.4)	−1.7 (−2.5, −0.9)	−3.3 (−4.5, −2.2)	−5.4 (−10.1, −0.8)	
Age 50–64 years, *n* (%)	1,123 (64)	209 (45)	702 (40)	143 (26)	5 (20)	***
Age 65–74 years, *n* (%)	397 (23)	143 (31)	536 (30)	212 (39)	11 (44)	
Age >75 years, *n* (%)	223 (13)	109 (24)	521 (30)	189 (35)	9 (36)	
Female, *n* (%)	929 (53)	240 (52)	994 (57)	308 (57)	15 (60)	***
Male, *n* (%)	814 (47)	221 (48)	765 (43)	236 (43)	10 (40)	
Most deprived, *n* (%)	220 (12)	70 (15)	354 (20)	119 (22)	7 (28)	***
Mid deprived, *n* (%)	1,077 (62)	274 (60)	1,002 (57)	321 (59)	13 (52)	
Least deprived, *n* (%)	446 (26)	117 (25)	403 (23)	104 (19)	5 (20)	

*Abbreviations*: CI, confidence interval; PCS, Physical Component Summary; SD, standard deviation.

***P < 0.01.

a Compared with non-chronic morbidity and adjusted for age, gender, and deprivation.

## 4 Discussion

This study has used LCGA to identify five clusters of people with different multimorbidity trajectories in primary care in older adults. A non-chronic morbidity cluster (40%) included those who did not have any chronic morbidity. An onset chronic morbidity cluster (10%) included people who developed a first chronic morbidity during the 3 years. People in the newly-developing multimorbidity cluster (37%) tended to develop multimorbidity over time. The evolving multimorbidity cluster (12%) included people with increasing counts of chronic diseases over time. People in the multi-chronic multimorbidity cluster (1%) were similar to those in the evolving cluster in that they developed further chronic diseases; however, in contrast to the multi-chronic cluster, those in the evolving cluster were less likely to start with multimorbidity.

Testing of these clusters in a second data set added further support to the existence of these multimorbidity trajectories. To our knowledge, no previous studies using LCGA have tested whether the trajectories identified also occur in another data set. Self-reported health status worsened with increasing severity of multimorbidity trajectories. Respondents in the non-chronic cluster reported the best general health and those in the multi-chronic multimorbidity cluster experienced the worst health status. This study has highlighted that half of this elderly population has more than one chronic morbidity, with many of those with multimorbidity continuing to develop further chronic problems over the course of 3 years. Clinicians need to recognize that chronic disease populations experience different morbidities and accrual of comorbidity over time may be different, and the type of morbidity may determine trajectories of the patient experience. The identified trajectories can also help in prognosis studies to help identify earlier and then target patients more at risk of developing more severe multimorbidity trajectories and hence the poorer health and outcomes associated with these trajectories. One potential application of understanding development of multimorbidity trajectories is within specific index patients. For example, within cardiovascular or musculoskeletal patients, the different trajectories applied could provide information on illness course and outcomes. Distinct trajectories then provide a method for differentiating levels of risk which may be amenable to different intervention approaches.

Our study confirmed the potential of using a large administrative data set such as primary care consultation records to identify multimorbidity. In our study, two-thirds of people aged >50 years consulted for at least 1 of the 42 chronic problems over 3 years and more than half developed further chronic problems, with corresponding poorer physical and mental health status, highlighting the impact of development of new chronic conditions. This shows the extent of chronic multimorbidity in older people in primary care, with previous studies reporting that between 55% and 98% of older adults have multimorbidity [11], [15], [24].

The lack of prospective studies of multimorbidity has been highlighted recently indicating that further research is needed in this area [10], [11], [12]. Existing prospective multimorbidity studies have defined multimorbidity as new morbidities which occurred any time during the whole follow-up period in addition to baseline morbidity and have not tried to identify different trajectories of multimorbidity [12]. One review further suggested that future research should study onset and change in multimorbidity [11]. A few studies have sought to define multimorbidity using multiple time points (more than two time points) and found that a change in multimorbidity status over time led to worse health outcomes [15], [38], [39], [40]. We have extended their findings to show that different pathways of developing multimorbidity have different associations with physical health and also found an association of these pathways with mental health. Our findings also echo an American study where people accumulated chronic morbidities at different rates over time [41]. However, multimorbidity in their study was based on seven self-reported morbidities and may be less accurate than morbidities recorded in primary care databases [13], [14].

Previous multimorbidity studies based on administrative data sets or using self-reported morbidity have applied approaches such as logistic regression, factor analysis, latent class analysis, and multilevel Bayesian networks to discover multimorbidity patterns [15], [23], [24], [25], [26], [27], [28]. Our findings are consistent with these studies, which, although these are often limited by examining only pairs of cooccurring diseases or based on a small selected pool of morbidities, showed common cooccurring combination of chronic morbidities that have well-established etiological relationships [15], [58], for example, diabetes mellitus and hypertension, hypertension and heart disease.

### 4.1 Strengths and weaknesses of this study

The use of LCGA in our study has overcome many of the limitations found in prior work such as identifying multimorbidity at a single point in time or focusing on a small number of morbidities. We were able to identify distinct longitudinal trajectories of chronic multimorbidity in primary care in older adults. These trajectories had different self-reported physical and mental health profiles. There are however extensions to latent class analysis that could have been used. For example, growth mixture modeling (GMM) assumes there are variations between people within clusters in morbidity counts around the cluster-specific mean count at each time point [50]. However, analyses not presented here showed that there was little variation within clusters and suggested nonidentification and convergence problems most likely because of use of count data in GMM models [50], [54], [59]. A further additional analysis using the three-step approach [60] explicitly incorporated age, gender, and deprivation as covariates in the model, while ensuring classification of the derived trajectories is most influenced by multimorbidity patterns rather than the covariates. This additional analysis suggested that explicit incorporation of covariates in our model did not have a significant impact on the multimorbidity trajectories identified, that is, multimorbidity trajectories are not just a proxy of age.

The current focus in this study was on a limited set of 42 chronic conditions; the full severity classification includes a range of other conditions, which have other dimensions of “severity,” such as whether conditions might be acute or life threatening. Although we found an association between the derived multimorbidity trajectories and self-reported psychological health, few mental health morbidities were included in our list of 42 chronic progressive conditions. Several (including anxiety and depression) were included in the wider starting pool of 188 morbidities but these tended to be classified in the consensus exercises as acute-on-chronic and recurrent conditions mainly because of their fluctuating symptoms presented. If these other morbidities or those used in other indices or studies were included (eg, the Charlson Comorbidity index [26] and Barnett's list which also included mental health problems [5]), it is possible that different trajectories may be generated. Further research could validate trajectories from a larger list of conditions, for example Barnett's list of 40 disease conditions [5]. Both data sets used in this study were local consultation data sets. Further research could assess whether the same trajectories of multimorbidity can be obtained in national primary care databases.

These trajectories provide better understanding of the course of multimorbidity in primary care and allow researchers to further investigate the development and prognosis of multimorbidity and its impact on both physical and mental health in primary care. We have developed lookup tables and a training data set (available from the lead author) to aid researchers allocate multimorbidity profiles based on counts of chronic conditions at six different time points in older people. This allows researchers to use any chronic condition recorded in their study, not just those included in our study. Ultimately, recognition of multimorbidity pathways may aid the case management process that focuses on providing primary care for older people who are likely to have a range of morbidities. There is increasing recognition that clinicians should move away from a single disease management approach in older adults and use an integral treatment or management which is indifferent from the biological age [5], [9], [10]. Future research also needs to incorporate such trajectories into identifying patients at risk of progression of disease and deterioration of health.
