# Supplementary material for: Distinct trajectories of multimorbidity in primary care were identified using latent class growth analysis
Source: J Clin Epidemiol. 2014 Oct;67(10):1163–71. doi: 10.1016/j.jclinepi.2014.06.003 (PMC4165436; doi:10.1016/j.jclinepi.2014.06.003)
Supplement: Appendix [file mmc1.docx]

**APPENDIX**

| List of Chronic Morbidities Based on Read Codes Included in the Study | | | | |
| --- | --- | --- | --- | --- |
| Read code^a^ | Read term | | Read code^a^ | Readterm |
| B7C | Benign neoplasm of prostate | | H31^d^ | Chronic bronchitis |
| B8z | Carcinoma in situ | | H32 | Emphysema |
| B92 | Neurofibromatosis - Von Recklinghausen's disease | | H35 | Extrinsic allergic alveolitis |
| C04 | Hypothyroidism | | H54 | Pulmonary oedema |
| C10 | Diabetes mellitus | | H56 | Diffuse pulmonary fibrosis |
| C32 | Pure hypercholesterolaemia | | K03 | Nephritis and nephropathy |
| C38 | Obesity | | K11 | Hydronephrosis |
| D10 | Hereditary haemolytic anaemia | | K20 | Prostatism |
| E00 | Senile/presenile dementia | | N04 | Rheumatoid arthritis |
| F36 | Hereditary and idiopathic peripheral neuropathy | | N05 | Osteoarthritis |
| F45 | Primary open-angle glaucoma | | N06 | Arthropathy |
| F46 | Senile cataract | | N11 | Cervical spondylosis |
| F4B | Corneal opacity and disorders of cornea | | N31 | Paget's disease of bone |
| F59 | Deafness | | N33 | Osteoporosis |
| G11 | Mitral stenosis | | SC2 | Late effect - nervous system injury |
| G1z | Rheumatic heart disease | | TPz | War injuries |
| G20^b^ | High blood pressure | |  |  |
| G21 | Hypertensive heart disease | |  |  |
| G22 | Hypertensive renal disease |  | |  |
| G33 | Angina pectoris |  | |  |
| G34^c^ | Ischaemic heart disease |  | |  |
| G57 | Atrial fibrillation |  | |  |
| G58 | Congestive heart failure |  | |  |
| G67 | Cerebral atherosclerosis |  | |  |
| G6z | Cerebrovascular disease |  | |  |
| G73 | Intermittent claudication |  | |  |

^a^ We identified morbidities collated at the third level of the Read Code hierarchy and thus included all codes under that code in the hierarchy; ^b^ also included Read Code recorded as “G2”; ^c^ also included Read Code recorded as “G3”; ^d^ also included Read Code recorded as “H3”
